# Bedside insertion of a peripherally inserted central catheter into a patient with BMI of 84.8 kg/m^2^ using a magnetic tracking and electrocardiogram-based tip confirmation system: a case report

**DOI:** 10.1186/s40981-022-00559-8

**Published:** 2022-08-27

**Authors:** Satoshi Uchida, Daiki Takekawa, Masaya Hori, Eiji Hashiba, Kazuyoshi Hirota

**Affiliations:** 1 Department of Anesthesiology, Hirosaki University Graduate School of Medicine, 5 Zaifu-cho, Hirosaki, 036-8562 Japan; 2 Division of Clinical Engineering, Hirosaki University Hospital, 53 Hon-cho, Hirosaki, 036-8563 Japan; 3 Division of Intensive Care, Hirosaki University Hospital, 53 Hon-cho, Hirosaki, 036-8563 Japan

**Keywords:** Peripherally inserted central catheter, Sherlock 3CG® Tip Confirmation System

## Abstract

**Background:**

Peripherally inserted central catheters (PICCs) are typically placed under fluoroscopy. We used a magnetic tracking and electrocardiogram-based tip confirmation system for insertion of a PICC insertion in a morbidly obese patient at the bedside.

**Case presentation:**

A 53-year-old female with severe obesity (height, 160 cm; weight, 217 kg; *BMI*, 84.8 kg/m^2^) was admitted to the intensive care unit. Both bilateral, inguinal, and cervical regions were covered with an excess of adipose tissue, making it difficult to place a central venous line. Since transferring her to fluoroscopy seemed dangerous, a PICC was inserted using Sherlock 3CG® TCS at the bedside. Magnetic sensor guidance failed due to the thick subcutaneous tissue her precordium, but intracavity electrocardiography could direct the tip to an appropriate position.

**Conclusion:**

We experienced bedside insertion of a PICC into a patient with BMI of 84.8 kg/m^2^ patient using a Sherlock 3CG® TCS. Since the interaction between Sherlock 3CG® TCS and body habitus has not been investigated, further reports are needed.

## Background

A peripherally inserted central catheter (PICC) is a type of venous catheter that is typically inserted with fluoroscopy. PICCs have less risk of procedure-related complications and bloodstream infections compared to other central venous catheters (CVCs), and their use has increased in recent years, especially in critical patients [1, 2]. The catheter tip is positioned at the cavoatrial junction (CAJ) through the subclavian and brachiocephalic veins, but it can sometimes be displaced. This leads to complications such as venous thrombosis or cardiac tamponade [3].

The Sherlock 3CG Tip Confirmation System (TCS, Bard Access Systems, Salt Lake City, UT, USA) is a device dedicated for insertion of PICCs. It is composed of a catheter with a magnetic sensor at the tip, a magnetic sensor placed on the chest wall, and a display showing the location and direction of the tip of the PICC as well as intracavity electrocardiogram (IC-ECG). This system has shown a high technical success rate [4]. Additionally, as Sherlock 3CG® TCS can be inserted without fluoroscopy, the risks associated with patient’s transfer may be avoided. We report bedside insertion of a PICC into a severely obese patient with a BMI of 84.8 kg/m^2^ using Sherlock 3CG® TCS.

## Case presentation

We obtained written informed consent from the patient for the publication of this case report. The patient was a 53-year-old female with severe obesity (height, 160 cm; weight, 217 kg; *BMI*, 84.8 kg/m^2^) and a past history of hypertension and diabetes mellitus. She had been febrile and in respiratory distress for several days and was treated with ceftriaxone and oxygen mask at another institution. Onset of CO_2_ narcosis with PaCO2 of 91.7 mmHg caused a deficit in consciousness, and her blood test showed renal dysfunction with inflammation. Computerized tomography showed a giant renal cyst, and urosepsis was suspected. She transferred to our institution to receive intensive care.

**Fig. 1:**
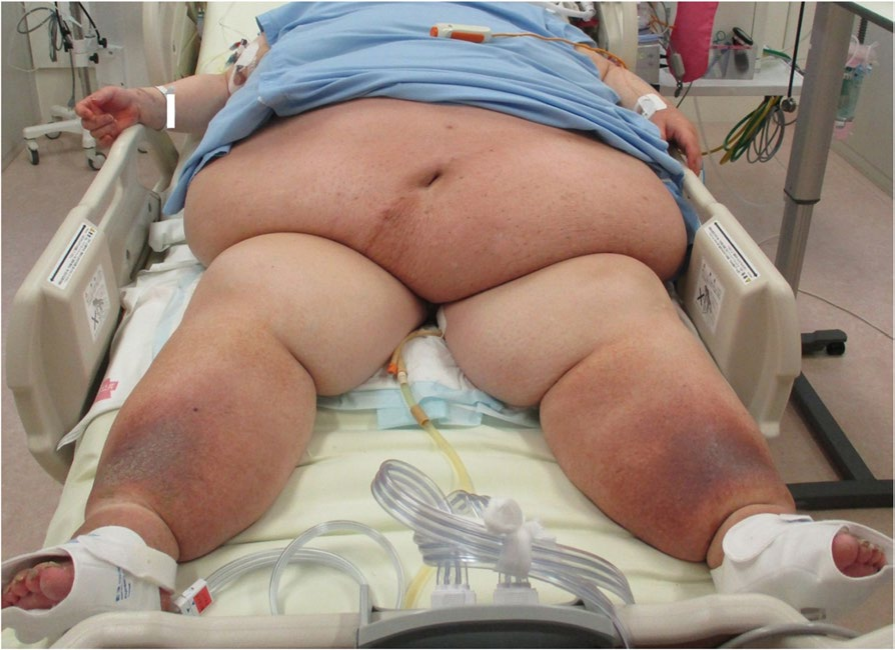
Bilateral inguinal regions covered with an excess of abdominal adipose tissue

**Fig. 2:**
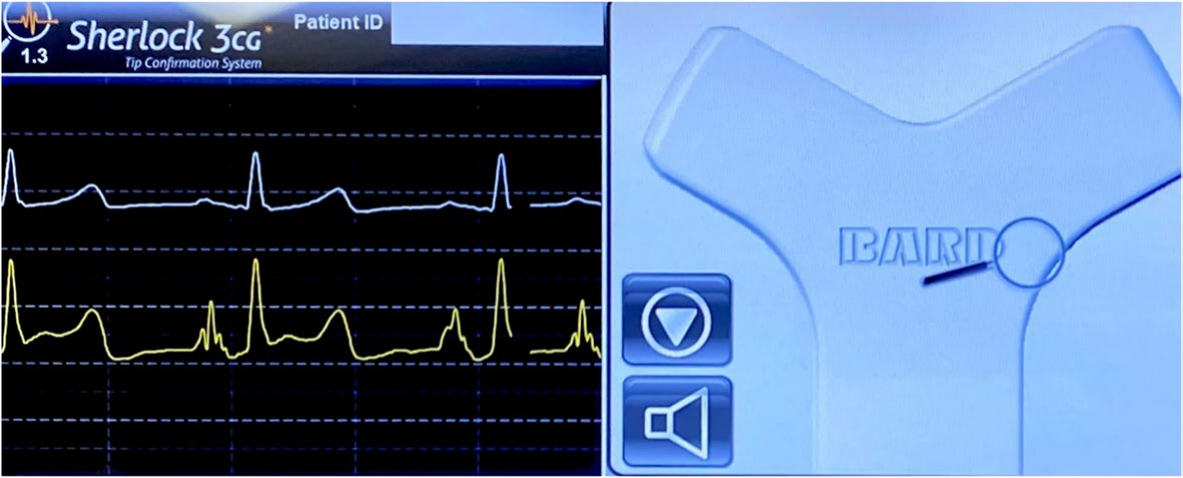
Body surface (left, top) and intracavity (left, down) electrocardiogram and an image on the display (right). Intracavity electrocardiogram was monitored via the peripherally inserted central catheter (PICC). Amplitude of the P waves is increased with advancing the PICC catheter and become highest with the tip located at the cavoatrial junction. Location of the tip of the PICC and the direction of the catheter are shown by a circle and a line, respectively, on the display

**Fig. 3:**
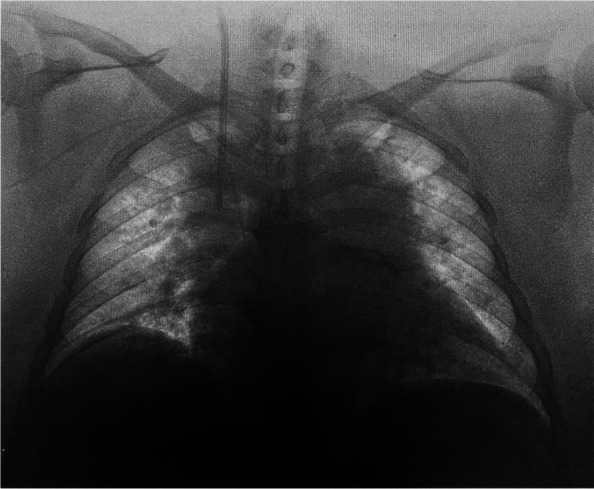
Exchange of the CVC and a vascular access line in the right internal jugular vein performed using a guide wire without any problem

The only venous route at the time of admission to the intensive care unit (ICU) was the CVC of the right internal jugular vein, and it was difficult to secure a peripheral venous route due to severe obesity. Renal replacement therapy was considered necessary for acute renal injury, but bilateral inguinal regions were covered with an excess of abdominal adipose tissue, making it difficult to place a vascular access line in the femoral vein (Fig. 1). We examined the insertion of a vascular access into the left internal jugular vein, but the subcutaneous tissue in the neck was also thick, making the procedure technically difficult. In addition, we feared pneumothorax or arterial puncture as a potentially fatal complication in this case. Furthermore, her orthopnea presented a challenge to bilateral internal jugular vein puncture. Therefore, we decided to place a PICC on the upper right arm using a Sherlock 3CG® TCS and to replace the CVC with a vascular access. The basilic vein was confirmed under echo guidance, and the sheath was successfully placed without any problem. We were able to pass the PICC without feeling any resistance, but the magnetic sensor did not display a tip icon on the monitor. It was detected intravascularly distal to the right internal jugular vein by ultrasonography. Thus, the PICC catheter was advanced while confirming the increase in the amplitude of P wave on IC-ECG, until negative (downward) deflection showing its passage through the CAJ appeared. The PICC was fixed at the position where the P wave was the highest (Fig. 2). Subsequent X-ray examination confirmed that the tip position was appropriate. Exchange of the CVC and a vascular access line in the right internal jugular vein was performed using a guide wire without any problem (Fig. 3).

The administration of meropenem and diuretics, and continuous hemodiafiltration, was started. Rehabilitation and dietary modification were performed in parallel, and her body weight was reduced by about 20 kg in 6 days. Despite her residual hypercapnia due to obesity hypoventilation syndrome, she was discharged from the ICU 7 days after admission because her general condition had improved. She was later transferred to her previous institution for weight loss intervention.

## Discussion

We present bedside insertion of a PICC into a patient with BMI 84.8 kg/m^2^ using a Sherlock 3CG® TCS. PICC is suitable for critically ill patients due to its advantages over CVC, including less risk of procedure-related complications and bloodstream infection [5]. A PICC is generally inserted via a cephalic or basilic vein of the upper arm, but it is sometimes displaced due to the long detention distance, so malposition of the catheter tip is one of the most common complications [6, 7]. Therefore, PICC is generally placed while being confirmed under fluoroscopic visualization. However, since it was a risk for this case to be transferred from the ICU, a PICC was inserted at the bedside using Sherlock 3CG® TCS.

The Sherlock 3CG® TCS is composed of an external magnetic sensor at the catheter tip and an IC-ECG guidance system. The magnetic sensor guidance system graphically shows the catheter tip on a bedside monitor. Therefore, catheter malposition can be recognized at an early stage if it strays into the right internal jugular vein or left innominate vein. As the catheter tip advances into the inferior vena cava, the P wave of the IC-ECG increases as it approaches the CAJ. A negative deflection appears in the P wave if the catheter tip passes through the CAJ, so the PICC should be placed at the highest P wave which is the appropriate insertion distance. Combination of these two systems present capacity to be inserted PICCs without using fluoroscopy [4].

The detectable depth of the magnetic sensor is 3–11 cm according to the standard limit. In our case, the distance from the chest wall to the CAJ was about 15 cm on the computerized tomography scan. Additionally, when a patient’s chest is not flat, the sensor will rest at an angle, causing an effect known as parallax. The difference between the point of view of the sensor and the user can be several centimeters. Therefore, in our case, the magnetic guidance system did not work due to the thick and angled subcutaneous tissue in the precordium. On the other hand, since the IC-ECG had worked accurately, we placed the catheter tip where the P wave is highest. It has been reported that an X-ray for confirmation when using Sherlock 3CG® TCS is unnecessary [8], but we confirmed that the tip position by the chest X-ray because the magnetic sensor did not worked. Success rates have not been reported when either the magnetic guidance or the IC-ECG failed to work properly, but in this case, the catheter tip was placed in the appropriate position.

## Conclusion

We experienced bedside insertion of a PICC into a patient with BMI 84.8 kg/m^2^ using the Sherlock 3CG® TCS. This device may be particularly useful in critical patients as it can reduce the risk associated with patient’s transfer. The depth of her subcutaneous precordial tissue disabled the function of the magnetic monitor, but an IC-ECG guidance system made it possible to place the tip in an appropriate position. The interaction between the Sherlock 3CG® TCS and severe obesity has not been investigated, and further research on its interaction with body habitus is needed.

## Data Availability

Not applicable.
